# Neoadjuvant Immunotherapy Effectiveness in Patients With Microsatellite Instability-High (MSI-H) Gastric Cancer

**DOI:** 10.7759/cureus.61344

**Published:** 2024-05-30

**Authors:** Viacheslav A Chubenko, Alexander Y Navmatulya, Ivan A Gerk, Artem A Sarmatov, Vitaliy V Egorenkov, Ksenia A Shelekhova, Evgeny N Zykov, Vera V Chernobrivceva, Nikita M Volkov, Vladimir M Moiseyenko

**Affiliations:** 1 Department of Chemotherapy, Napalkov State Budgetary Healthcare Institution, Saint Petersburg Clinical, Scientific, and Practical Center for Specialised Types of Medical Care (Oncological), Saint Petersburg, RUS; 2 Department of Abdominal Surgery, Napalkov State Budgetary Healthcare Institution, Saint Petersburg Clinical, Scientific, and Practical Center for Specialised Types of Medical Care (Oncological), Saint Petersburg, RUS; 3 Department of Surgery, Napalkov State Budgetary Healthcare Institution, Saint Petersburg Clinical, Scientific, and Practical Center for Specialised Types of Medical Care (Oncological), Saint Petersburg, RUS; 4 Department of Pathology, Napalkov State Budgetary Healthcare Institution, Saint Petersburg Clinical, Scientific, and Practical Center for Specialised Types of Medical Care (Oncological), Saint Petersburg, RUS; 5 Department of Radioisotope, Napalkov State Budgetary Healthcare Institution, Saint Petersburg Clinical, Scientific, and Practical Center for Specialised Types of Medical Care (Oncological), Saint Petersburg, RUS; 6 Department of Radiology, Napalkov State Budgetary Healthcare Institution, Saint Petersburg Clinical, Scientific, and Practical Center for Specialised Types of Medical Care (Oncological), Saint Petersburg, RUS; 7 Department of Medical and Radiation Therapy, Napalkov State Budgetary Healthcare Institution, Saint Petersburg Clinical, Scientific, and Practical Center for Specialised Types of Medical Care (Oncological), Saint Petersburg, RUS; 8 Department of the Director, Napalkov State Budgetary Healthcare Institution, Saint Petersburg Clinical, Scientific, and Practical Center for Specialised Types of Medical Care (Oncological), Saint Petersburg, RUS

**Keywords:** monotherapy, gastric cancer, msi-h, neoadjuvant, immunotherapy

## Abstract

Purpose

This research work evaluates monotherapy with checkpoint inhibitors (CPI). as a neoadjuvant treatment for patients with Microsatellite Instability-High (MSI-H) locally advanced gastric cancer.

Methods

Here we present the results of the retrospective study from Napalkov Cancer Center over 4.5 years on patients with MSI-H locally advanced gastric cancer. A total of 566 patients were analyzed, 18 of whom were included in the research, focusing on clinical response rate, surgical pathology, ‘watch and wait’ strategy, and safety outcomes on an exploratory basis. Patients were assigned to four to eight neoadjuvant cycles of CPI, followed by surgery.

Results

The objective response to neoadjuvant CPI in patients with MSI-H gastric cancer was 77.8%. Complete response was achieved in five (27.8%) and partial response in nine (50%) patients, accordingly. Surgery was performed on 14 patients. Complete margin-free (R0) resection rates were 100%. Downstaging was observed in 12 out of 14 patients. Histopathologic complete response rates (pathologic complete response or Tumor Regression Grade-major response (TRG1)) were achieved in eight (57.1%) patients. No disease progression was detected with a median follow-up of 33.7 months (4.4-55.7 months). Clinically significant adverse events were not observed.

Conclusion

CPI in a neoadjuvant setting for patients with MSI-H locally advanced gastric cancer is highly effective and safe.

## Introduction

Currently, gastric cancer ranks among the leading morbidity and mortality causes worldwide [1]. Unfortunately, 5-year survival in patients after surgical treatment does not exceed 20-30%, with a recurrence risk reaching 80% [2]. In this regard, the standard treatment strategy for locally advanced gastric cancer involves the usage of perioperative chemotherapy with surgery, which can achieve a 5-year survival rate of 45% [3]. On the other hand, molecular and biological subtypes of stomach cancer are actively researched with the aim of personalized selection of various treatment options. The Cancer Genome Atlas (TCGA) classified gastric cancer into four groups: EBV (Epstein-Barr virus) - 9%, MSI (microsatellite instability) - 21%, CIN (chromosomal instability) - 20%, and GS (genomically stable) - 50% [3]. One of the subtypes is characterized by defects in the mismatch repair genes (Microsatellite Instability-High (MSI-H)). According to literature data, its frequency ranges from 1% to 32% depending on various factors such as geographic region, localization of the primary tumor, histological subtype, age, and gender of the patients [4]. Overall, this subtype is characterized by a favorable prognosis, low risk of disease recurrence, and early-stage diagnosis [5]. However, this tumor subtype has low sensitivity to cytostatic drugs, which necessitates the search for pathogenetic treatment strategies [6]. One of these strategies is the usage of checkpoint inhibitors due to the higher tumor immunogenicity [7]. To date, checkpoint inhibitors demonstrate high efficacy in various solid tumors. In the case of MSI-H metastatic tumors, the objective response rate reaches more than 50% [3]. The combination of checkpoint inhibitors (CPI) with chemotherapy is included in the standards of care for patients with metastatic gastric cancer, leading to increased frequency of objective response and progression-free survival (with Combined Positive Score (CPS)≥ 5) [5]. Undoubtedly, their effectiveness in the neoadjuvant setting in patients with locally advanced disease is of interest, given the presence of a predictive biomarker, potential achievement of complete pathomorphological response, low clinical significant toxicity, and impact on overall survival. The aim of this study is to investigate the effectiveness of neoadjuvant monotherapy in patients with locally advanced MSI-H gastric cancer.

## Materials and methods

A retrospective analysis of the medical records from the information system of Napalkov State Budgetary Healthcare Institution, Saint Petersburg Clinical, Scientific, and Practical Center for Specialised Types of Medical Care (Oncological) was conducted from June 2019 to December 2023 (4.5 years). Eligible criteria for patients in the study were locally advanced (stage II-III) gastric cancer or gastroesophageal junction cancer (GC/GEJ) and evidence of defects in the mismatch repair genes (microsatellite instability-high (MSI-H)) detected by immunohistochemistry or molecular-genetic methods. All patients provided voluntary informed consent before the study began. Statistical analysis was performed using SPSS Statistics (IBM Corp., Armonk, USA).

**Figure 1:**
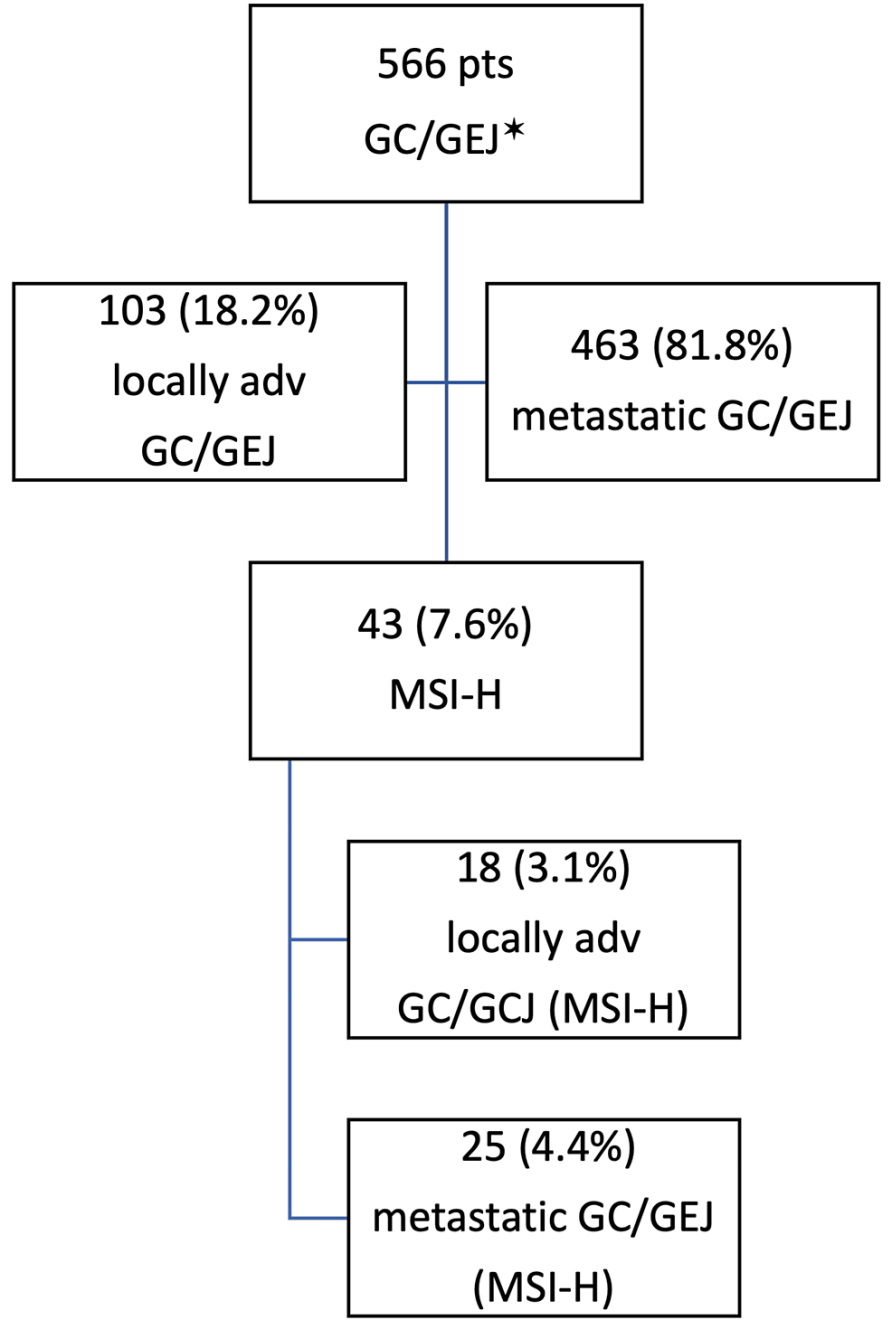
Flow-chart of the patients in the study *GC/GEJ - gastric cancer/gastroesophageal junction cancer; locally adv - locally advanced; MSI-H - microsatellite instability-high; pts - patients.

A total of 566 patients who underwent testing for defects in the mismatch repair genes were analyzed. Among them, 103 (18.2%) were diagnosed with locally advanced disease. MSI-H was detected in 43 (7.6%) patients. Among them, 18 (41.8%) had locally advanced disease and were included in the study (Figure 1).

All patients underwent the esophagogastroscopy with biopsy, CT, or PET-CT scan - five (27.8%) and 13 (72.2%) patients, respectively, and diagnostic laparoscopy and peritoneal lavage with cytological examination (12 out of 18 patients) before the treatment onset. 

After preoperative therapy, follow-up examinations (CT or PET-CT scan, esophagogastroscopy, and repeated biopsy) were performed. The next step was surgical treatment. At the time of analysis, 14 out of 18 patients (77.8%) had undergone surgery, while four patients (22.2%) refused. The post-surgery therapeutic pathomorphosis degree was evaluated by the Ryan scale. 

Subsequently, all patients were scheduled for follow-up with examinations every 3 months.

## Results

Characteristics of patients included in the study are presented in Table 1. In nine (50%) of the patients, MSI-H was determined by immunohistochemistry, while in the remaining cases, the polymerase chain reaction (PCR) method was used.

**Table 1:** Baseline patient and tumor characteristics. GEJ - gastroesophageal junction cancer; GC: gastric cancer

Data	N (%)
Sex	
Male	11 (61.1%)
Female	7 (38.9%)
Age, median	65 (56-81)
Diagnosis	
GEJ	3 (16.7%)
GC, body	8 (44.4%)
Distal GC	7 (38.9%)
Stage	
IIA	2 (11.1%)
IIB	13 (72%)
IIIA	3 (16.7%)
T clinical	
Т2	1 (5.6%)
Т3	14 (77.8%)
Т4а	2 (11.1%)
Т4b	1 (5.6%)
N clinical	
N0	3 (16.7%)
N1	15 (83.3%)

As a preoperative treatment, all patients received immunotherapy. The treatment regimens are presented in Table 2.

**Table 2:** Preoperative therapy in patients included in the study. Nivolumab – 3 mg/kg or 240 mg IV on day 1; pembrolizumab 200 mg IV on day 1 Cycles, median – 5.5 (4-8) Cycles, no surgery, median (N=4) – 22.3 (9-37)

Regimen (N=18)	N (%)
Nivolumab	11 (61.1%)
Pembrolizumab	6 (33.3%)
Nivolumab/Pembrolizumab	1 (5.6%)
Number of cycles before surgery (N=14)	N (%)
4	6 (42.9%)
6	5 (35.7%)
7	1 (7.1%)
8	2 (14.3%)

Adjuvant treatment was prescribed in the postoperative period. The treatment regimens are presented in Table 3.

**Table 3:** Adjuvant therapy regimens for patients included in the study. XELOX - oxaliplatin 130 mg/m2 IV on day 1, capecitabin 2000 mg/m2 d1-14 p.o.) FLOT (oxaliplatin 85 mg/m2 IV on day 1, docetaxel 50 mg/m2 IV on day 1, leucovorin 200 mg/m2 IV on day 1, 5-FU 2600 mg/m2 cont. inf. over 24 hours on day 1) FOLFOX (oxaliplatin 85 mg/m2 once every 14 d IV, leucovorin 200 mg/m2 d1-2 IV., 5-FU 400 mg/m2 bolus IV, 5-FU 2400 mg/m2 46 h cont. inf. IV) Nivolumab – 240 mg once every 14 d IV Number of cycles: 3 pts – XELOX №2; 1 pt – XELOX №1; XELOX №4; FLOT №1; FOLFOX №1; nivolumab №3

Regimen (N=14)	N (%)
XELOX	5 (35.7%)
FLOT	1 (7.1%)
FOLFOX	1 (7.1%)
Nivolumab	1 (7.1%)

The effectiveness of preoperative immunotherapy is presented in Table 4.

**Table 4:** The effectiveness of preoperative immunotherapy in patients with MSI-H locally advanced gastric cancer included in the study. CR – complete response; PR – partial response; SD – stable disease; PD – progressive disease; ND – not done.

Objective response (N=18)	N (%)
PET-CT scan	
CR	5 (27.8%)
PR	9 (50%)
SD	3 (16.7%)
PD	1 (5.6%)
Esophagogastroscopy	
CR	7 (38.9%)
PR	5 (27.8%)
SD	2 (11.1%)
PD	0
ND	4 (22.2%)

During the preoperative immunotherapy in patients with MSI-H locally advanced gastric cancer, an objective response was achieved in 14 pts (77.8%). Stable of the disease was observed in three patients and disease progression was noted in one patient. It is noteworthy that no correlation was found between the results of radiological and endoscopic examinations. In one patient, there was an increase in metabolic activity in the primary tumor observed on PET-CT. However, clinically and based on endoscopic data, positive dynamics were noted, including regression of the pain in the epigastric area and reduction in the size of the primary tumor, respectively.

Out of 18 patients, 14 (77.8%) underwent surgery. All operated patients achieved R0 resection. Assessment of the tumor pathomorphological response was performed using the Ryan scale. As a result of the preoperative immunotherapy, a complete pathological response (pCR) was observed in six patients (42.9%). A near-complete pathological response was detected in two (14.3%) patients The degree of therapeutic pathomorphosis is presented in Table 5. The pathomorphological stage changed in all patients receiving preoperative immunotherapy. Primary tumor size reduction was observed in 12 patients (85.7%). In two cases (14.3%), the size of the primary tumor increased and corresponded to ypT4b. In another case, an increase in the size of the lesion on PET-CT scan was accompanied by a change in the disease stage from cT3N0 to ypT2N1. It is important to note that regional lymph node involvement persisted in four (28.5%) patients. Thus, we did not observe a correlation between the metabolic and pathomorphological response of the tumor to treatment.

**Table 5:** Pathologic tumor regression in operated patients after preoperative immunotherapy. TRG - Tumor Regression Grade; ypTNM - Pathologic Tumor-Node-Metastasis

Grade (N=14)	N (%)
Ryan degree	
TRG0	6 (42.9%)
TRG1	2 (14.3%)
TRG2	5 (35.7%)
TRG3	1 (7.1%)
ypTNM	
ypT0N0	6 (42.9%)
ypT1aN0	2 (14.3%)
ypT1bN0	1 (7.1%)
ypT2N0	1 (7.1%)
ypT0N1	1 (7.1%)
ypT2N1	1 (7.1%)
ypT4bN1	1 (7.1%)
ypT4bN3	1 (7.1%)

The overall characteristics of the patients are presented in Table 6.

**Table 6:** Summary of included patients during the preoperative immunotherapy. Out of 18 patients, 14 underwent surgery. Complete and near-complete pathomorphological response was observed in 57.2%. Four patients are under observation without signs of disease progression. Two patients died as a result of an infectious disease and concomitant disease unrelated to the gastric cancer. CR – complete response; PR – partial response; SD – stable disease; PD – progression disease; ND – not done; cM - Clinical Tumor-Node-Metastasis; EGD - Esophagogastroduodenoscopy; ypTNM - Pathologic Tumor-Node-Metastasis

№	Age, y	cTNM	Neoadjuvant phase	PET-CT	EGDs	ypTNM	TRG	Adjuvant phase	Censor	Follow-up, months
1	63	cT3N1	Nivolumab 6 cycles	PR	CR	ypT0N0	TRG0	Nivolumab 3 cycles	No	55.7
2	72	cT3N1	Nivolumab 8 cycles	CR	CR	ypT0N1	TRG1	-	Comorb	32.7
3	64	cT3N1	Nivolumab 4 cycles	PR	PR	ypT0N0	TRG0	XELOX 2 cycles	No	46.5
4	71	cT3N1	Pembrolizumab 4 cycles	PR	PR	ypT1bN0	TRG2	XELOX 4 cycles	No	46.4
5	65	cT4aN1	Pembrolizumab 4 cycles	CR	PR	ypT1aN0	TRG2	XELOX 2 cycles	No	47.4
6	59	cT3N1	Pembrolizumab 4 cycles	PR	ND	ypT1aN0	TRG1	FLOT 1 cycle	No	40.4
7	73	cT3N0	Pembrolizumab 4 cycles	PR	PR	ypT0N0	TRG0	XELOX 1 cycle	No	46.8
8	68	cT4aN1	Pembrolizumab 4 cycles	SD	CR	ypT0N0	TRG0	XELOX 2 cycles	No	41.1
9	57	cT3N0	Nivolumab 8 cycles	PD	PR	ypT2N1	TRG2	FOLFOX 1 cycle	COVID	12.6
10	67	cT3N1	Pembrolizumab 6 cycles	PR	ND	ypT4bN1	TRG2	-	No	10.5
11	56	cT3N1	Nivolumab 3 cycles, Pembrolizumab 3 cycles	CR	CR	ypT0N0	TRG0	-	No	9.2
12	65	cT3N1	Nivolumab 7 cycles	SD	SD	ypT4bN3	TRG3	-	No	4.9
13	68	cT4bN0	Nivolumab 6 cycles	PR	CR	ypT0N0	TRG0	-	No	4.3
14	57	cT3N1	Nivolumab 6 cycles	PR	PR	ypT2N0	TRG2	-	No	4.9
15	56	cT3N1	Nivolumab 30 cycles	CR	CR	-	-	-	No	49.6
16	57	cT3N1	Nivolumab 37 cycles	CR	CR	-	-	-	No	34.6
17	73	cT3N1	Nivolumab 13 cycles	PR	ND	-	-	-	No	8.0
18	81	cT2N1	Nivolumab 9 cycles	SD	ND	-	-	-	No	9.3

**Figure 2:**
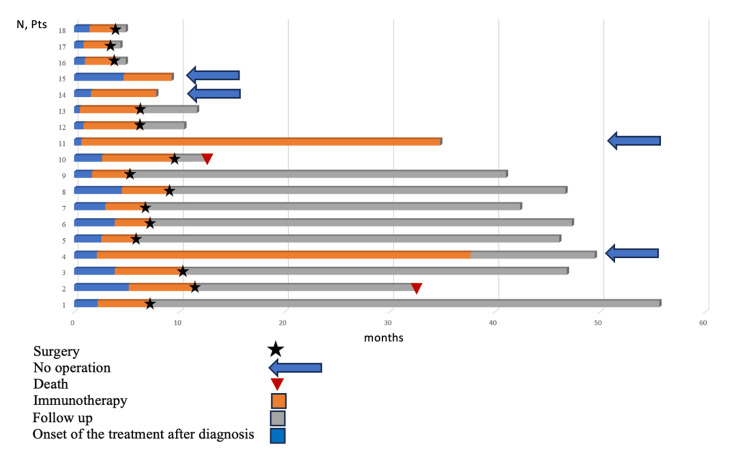
Duration of response in patients during preoperative immunotherapy. pts - patients

**Figure 3:**
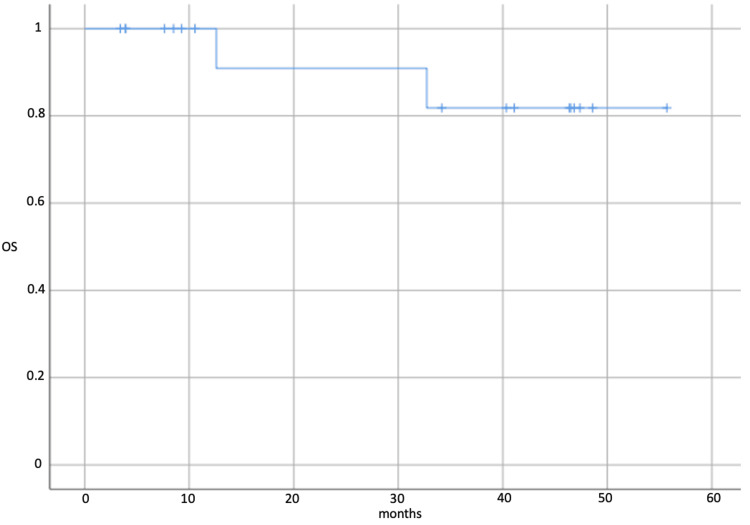
Kaplan-Meier plot of overall survival in patients during the preoperative immunotherapy. OS - overall survival

Nowadays, the median follow-up in the study is 33.7 months (ranging from 4.4 to 55.7 months). Disease progression was not registered (Figure 2). Two patients died as a result of concomitant cardiac pathology and the novel coronavirus disease infection (COVID-19). The median overall survival has not been reached (Figure 3). The 3-year overall survival rate was 88.9%.

Special interest is represented by four (22.2%) patients who did not undergo surgical treatment. Two achieved complete and one achieved partial metabolic response, and one achieved stable disease according to PET-CT data. Among the two patients who showed complete response on PET-CT scan, one had no tumor detected on esophagogastroduodenoscopy, and the other had no tumor cells detected on repeat biopsy at the site of the primary tumor. Immunotherapy was discontinued after 30 infusions in one of them due to achieving maximum effect. He continues to be monitored without signs of disease progression for 11 months to date. The others continue therapy (Figure 2).

Despite long-term use of the immunotherapy, no clinically significant immune-mediated toxicity was detected. One patient reported autoimmune hyperthyroidism with grade 2.

## Discussion

The study's comprehensive analysis underscores the potential of preoperative immunotherapy in patients with locally advanced gastric cancer characterized by MSI-H [3]. The observed outcomes, including significant objective response rates and disease stabilization, suggest a promising avenue for further exploration and clinical application of immunotherapy in this setting. These findings emphasize the importance of continued research efforts to elucidate the role of immunotherapy in the management of gastric cancer, highlighting the need for tailored treatment approaches and ongoing monitoring to optimize patient outcomes [4-6]. 

MSI-H gastric and gastroesophageal junction tumors represent a distinct type of disease characterized by high mutational burden and severe immune infiltration [5]. These data provide a basis for research immunotherapy in patients with advanced, chemotherapy-refractory tumors. To date, a high frequency of objective responses (up to 57%) and prolonged survival are demonstrated when checkpoint inhibitors are prescribed in this population [8].

As for locally advanced gastric cancer, the standard approach is perioperative chemotherapy followed by surgical treatment [9, 10, 11]. However, the appropriateness of this strategy in MSI-H patients is not always justified. This is explained by the fact that this type of tumor has a better prognosis [9], but a lower frequency of Pathologic complete response(pCR), as demonstrated with ECF chemotherapy (epirubicin, cisplatin, 5-fluorouracil) [10]. Meanwhile, perioperative chemotherapy with the FLOT regimen (oxaliplatin, paclitaxel, 5-fluorouracil) resulted in a 16% pCR in the overall population [11] and 42% in the MSI-H population [12]. However, treatment regimens involving cytostatics are highly toxic, necessitating the development of new precision therapy methods for this patient’s subgroup [12].

Neoadjuvant immunotherapy has demonstrated high efficacy in skin melanoma and lung cancer treatment. Currently, its effectiveness is known in MSI-H locally advanced rectal and colon cancer [13, 14]. The rationale for early prescription of checkpoint inhibitors in resectable disease is the increased number of neoantigens and tumor-infiltrating lymphocytes, as well as the potential for memory cell formation, which theoretically can prevent disease recurrence [4, 5]. Therefore, examination of neoadjuvant immunotherapy in patients with locally advanced MSI-H gastric cancer is of significant interest [8].

According to the literature, in the past 5 years, 13 studies including checkpoint inhibitors in neoadjuvant therapy regimens for gastric cancer have been published [12]. However, only four of them conducted subgroup analysis in the MSI-H population, and two of them investigated monotherapy [15-18].

In our analysis, a group of patients with locally advanced gastric cancer and MSI-H were treated with neoadjuvant monoimmunotherapy. The median follow-up for this population was 33.7 months, which is the longest reported in the literature.

In the phase II GERCOR NEONIPIGA study, 27 patients received neoadjuvant immunotherapy with ipilimumab+nivolumab, the median follow-up was 14.9 months. In this study, a pCR was observed in 58.6% of patients, while the frequency of grade 3-4 adverse events was 19%, likely due to the combination of checkpoint inhibitors [15]. In our analysis, with monotherapy, a pCR was achieved in 42.9% of patients, and no grade 3-4 adverse events were reported.

Although complete pathologic response is a surrogate marker for survival in locally advanced disease, our study did not find its correlation [15]. None of the patients, regardless of postoperative Pathologic Tumor-Node-Metastasis​​​​​​​ (ypTNM) stage or Tumor Regression Grade​​​​​​​ (TRG)pathologic response according to Ryan's classification, showed disease progression during the long follow-up period (over 3 years).

Currently, most published clinical studies on gastric cancer analyze the effectiveness of combination therapy in the neoadjuvant regimen, which includes chemotherapy and checkpoint inhibitors [2, 8, 12, 18]. It is expected that such a combination will increase the frequency of complete pathologic tumor response and impact patient survival. However, the 10% difference in achieving pCR, the lack of influence on disease-free survival, and the increase in clinically significant toxicity raise doubts about the choice of this approach in treating patients with locally advanced MSI-H gastric cancer [12].

Further research is needed to determine the optimal assessment of the tumor response to immunotherapy [17]. Contrast-enhanced computed tomography does not always accurately characterize the dynamics of lesions in hollow organs during treatment [16, 17]. PET-CT allows the evaluation of tumor metabolism in addition to size [17]. However, our study showed that PET-CT results sometimes do not correspond to the endoscopic findings and do not predict pathologic response. According to the literature, the concordance between instrumental and pathologic response is only 49%.

Additionally, our study did not determine the optimal number of immunotherapy cycles before surgery. Some ongoing studies propose a short course of immunotherapy, such as two cycles, as seen in the GERCOR NEONIPIGA study, or prolonged treatment for 6 months [15]. We prescribed four to eight cycles of immunotherapy before surgery, which is standard practice for perioperative chemotherapy, but did not observe differences in treatment outcomes.

Of particular interest in our study are the patients who did not undergo surgical treatment. Currently, there are already known groups of patients with MSI-H rectal cancer for whom a watch-and-wait approach is applied [14, 16, 17]. In our analysis, we presented a similar approach for patients with gastric tumors for the first time. The median follow-up in this group of patients (n=4) was 22.0 months, and most importantly, no disease progression was observed during this time. The optimal strategy for managing patients with potentially resectable tumors requires further investigation in terms of survival duration and the search for surrogate markers of complete pathologic tumor regression [18].

Limitations of this study include its retrospective nature, the use of different immunotherapeutic agents, varying numbers of treatment cycles, and the prescription of chemotherapy to some patients after surgery. Nevertheless, our analysis reflects real-world clinical practice and demonstrates the high efficacy of monoimmunotherapy in patients with locally advanced MSI-H gastric cancer. The following unanswered questions remain - the number of neoadjuvant cycles, the need for adjuvant therapy considering the effect of neoadjuvant immunotherapy, and the duration of immunotherapy in cases of complete regression (including pathologic regression) when patients refuse surgery.

## Conclusions

The study's comprehensive analysis underscores the potential of preoperative immunotherapy in patients with locally advanced gastric cancer characterized by MSI-H status. The observed outcomes, including significant objective response rates and long disease stabilization, suggest a promising avenue for further exploration and clinical application of immunotherapy in this setting. These findings emphasize the importance of continued research efforts to elucidate the role of immunotherapy in the management of gastric cancer, highlighting the need for tailored treatment approaches and ongoing monitoring to optimize patient outcomes.
